# The Dietary Replacement of Soybean Oil by Canola Oil Does Not Prevent Liver Fatty Acid Accumulation and Liver Inflammation in Mice

**DOI:** 10.3390/nu12123667

**Published:** 2020-11-28

**Authors:** Marina Masetto Antunes, Guilherme Godoy, Ingrid de Lima Fernandes, Luciana Pelissari Manin, Caroline Zappielo, Laureane Nunes Masi, Vivian Araújo Barbosa de Oliveira, Jesuí Vergílio Visentainer, Rui Curi, Roberto Barbosa Bazotte

**Affiliations:** 1 Department of Pharmacology and Therapeutics, State University of Maringá, Maringá 87020-900, Brazil; antunes.mah@gmail.com (M.M.A.); godoy_guilherme@hotmail.com (G.G.); 2 Department of Chemistry, State University of Maringá, Maringá 87020-900, Brazil; ingrid.delima92@gmail.com (I.d.L.F.); lucianapmanin@hotmail.com (L.P.M.); carolzappi@hotmail.com (C.Z.); jesuiv@gmail.com (J.V.V.); 3 Interdisciplinary Post-Graduate Program in Health Sciences, Cruzeiro do Sul University, São Paulo 03342-000, Brazil; laure_masi@hotmail.com (L.N.M.); vivianbiomedicina@gmail.com (V.A.B.d.O.); curirui@gmail.com (R.C.)

**Keywords:** macronutrients, non-alcoholic fatty liver disease (NAFLD), fatty acids, cytokines

## Abstract

A high-carbohydrate diet (HCD) is a well-established experimental model of accelerated liver fatty acid (FA) deposition and inflammation. In this study, we evaluated whether canola oil can prevent these physiopathological changes. We evaluated hepatic FA accumulation and inflammation in mice fed with a HCD (72.1% carbohydrates) and either canola oil (C group) or soybean oil (S group) as a lipid source for 0, 7, 14, 28, or 56 days. Liver FA compositions were analyzed by gas chromatography. The mRNA expression of acetyl-CoA carboxylase 1 (ACC1) was measured as an indicator of lipogenesis. The mRNA expression of F4/80, tumor necrosis factor-α (TNF-α), interleukin (IL)-1β, IL-6, and IL-10, as mediators of liver inflammation, were also measured. The C group stored less n-6 polyunsaturated FAs (n-6 PUFAs) and had more intense lipid deposition of monounsaturated FAs (MUFAs), n-3 PUFAs, and total FAs. The C group also showed higher ACC1 expression. Moreover, on day 56, the C group showed higher expressions of the inflammatory genes F4/80, TNF-α, IL-1β, and IL-6, as well as the anti-inflammatory IL-10. In conclusion, a diet containing canola oil as a lipid source does not prevent the fatty acid accumulation and inflammation induced by a HCD.

## 1. Introduction

Hepatic lipid accumulation is the hallmark of non-alcoholic fatty liver disease (NAFLD), one of the most common diseases worldwide, which can progress to steatohepatitis, fibrosis, cirrhosis, and liver function failure [1]. NAFLD has also been associated with other disorders, such as obesity and cardiovascular diseases [2], and occurs due to an imbalance between the synthesis and exportation of lipids from the liver. The liver lipid accumulation occurs through increased dietary lipid intake, abnormal repartitioning of triacylglycerol (TAG) from adipose tissue to the liver, and increased de novo fatty acid (FA) synthesis and lipogenesis (DNL) [3].

Animals fed a stipulated diet of macronutrient composition provide experimental alternatives to overcome the limitation of studies in humans, where there is a large variability in the daily diet composition. In this context, we previously demonstrated that the diet for maintenance of laboratory adult rodents proposed by the American Institute of Nutrition (AIN-93-M) is more inflammatory and lipogenic than a high-fat diet in the liver of Swiss mice [4]. The carbohydrate content in the AIN-93 diet is 72.1% [5] versus 50–55% in the rodent chow diet [6]. Thus, the AIN-93-M diet can be considered a high-carbohydrate diet [4,7,8,9,10]. The n-3 polyunsaturated FAs (n-3 PUFAs) inhibit hepatic lipogenesis and inflammation, preventing NAFLD [11,12,13]. In contrast, higher liver levels of saturated FAs (SFAs), monounsaturated FAs (MUFAs), and elevated n-6:n-3 PUFA ratio have been associated with higher inflammation state and NAFLD occurrence [14,15,16].

In the Western diet, vegetable oil consumption as a source of FAs has been increasing [17,18,19]. One of the most consumed oils in the Western diet is canola oil, a cheaper substitute for olive oil. Canola oil, when compared to soybean oil, contains lower levels of SFAs and a lower n-6:n-3 PUFA ratio [19].

Canola oil regulates the lipid profile and can play protective roles against metabolic syndrome, cardiovascular disease risk, and renal dysfunction caused by type 1 diabetes [20,21,22,23,24,25,26,27,28,29].

Despite numerous studies, the potential beneficial effects of canola oil still need confirmation. For example, the lifespan has been shown to be shorter in stroke-prone spontaneously hypertensive rats fed canola oil as a sole lipid source than soybean oil [30,31,32,33,34]. One study has reported memory impairments and reduced synaptic integrity in a transgenic mouse model of Alzheimer’s disease [35]. Rats fed canola oil have shown insulin resistance [17] and higher blood pressure [36], compared to those fed with soybean oil. In addition, no study has compared the effect of canola oil and soybean oil based lipid intake on liver FAs’ deposition and inflammation.

A high-carbohydrate diet is a well-established experimental model of liver FA accumulation and inflammation [4,37,38]. Herein, we evaluate whether canola oil can prevent the liver FA deposition and inflammation induced by a high-carbohydrate diet. For comparative purposes, the reference group received soybean oil as the source of lipids.

## 2. Materials and Methods

### 2.1. Animals and Diets

The experimental protocol was approved by the Animal Ethics Committee of The State University of Maringá (CEUA).

We used male Swiss mice (six weeks old) receiving standard rodent chow (Quintia-Nuvilab^®^, Colombo city, Brazil) from weaning. The mice were individually housed and maintained at a controlled temperature (23 ± 1 °C), humidity (55 ± 10%), photoperiod (12 h light/12 h darkness), and had free access to water and food.

We prepared the diets with highly refined ingredients purchased from the Rhoster Company (Araçoiaba da Serra, SP, Brazil). The diet composition was based on purified diets for the maintenance of laboratory adult rodents proposed by the American Institute of Nutrition (AIN-93-M). The protein, carbohydrate, and total fat contents in the diets were 14.0, 72.8, and 4.0 g/100 g, respectively. The main FA compositions of the diets were measured (Table 1).

Carbohydrate composition was represented by cornstarch (46.6%), dextrinized cornstarch (15.5%), and sucrose (10.0%) [5]. As soybean oil is the source of lipids in the AIN-93 diet, the control group was represented by mice fed with the AIN-93 diet (high-carbohydrate diet) with soybean oil as a fat source (S group). The experimental group received the AIN-93 diet (high-carbohydrate diet) with canola oil as a fat source (C group).

**Figure 1:**
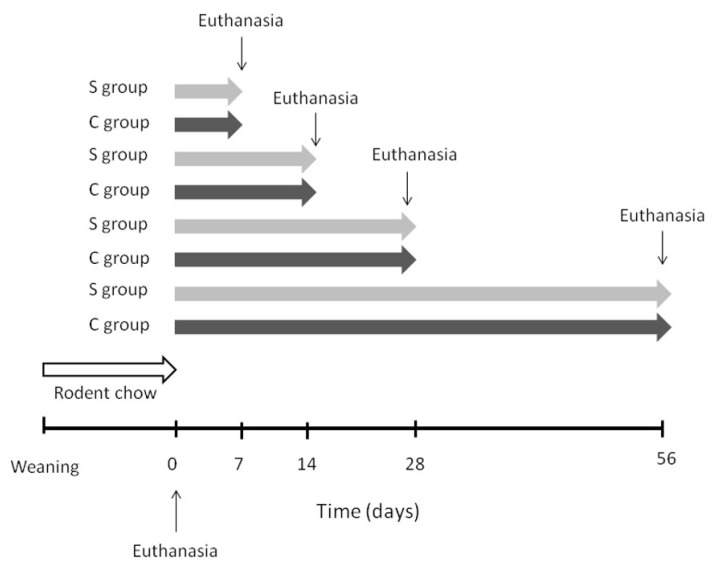
Treatment schedule: All mice received standard rodent chow from weaning and before starting the diets (time 0). The high-carbohydrate diet containing canola oil or soybean oil as a lipid source was administered for 0 (before starting the diets), 7, 14, 28, or 56 days.

After receiving the diets for 0 (before starting the diets), 7, 14, 28, or 56 days, the mice were fasted from 5:00 p.m. to 8:00 a.m. the following day and then euthanized. We measured blood glucose, TAG, and cholesterol concentrations, according to the manufacturer’s instructions. The livers were removed, weighed, and stored in liquid nitrogen until further analysis. The treatment schedule of the animals is shown in Figure 1.

### 2.2. Analysis of Liver Fatty Acid Composition

We used triturated samples (100 mg) from frozen livers to determine the total lipid content and FAs. We transesterified total lipids utilizing the method of Figueiredo et al. (2016) [39]. Methyl ester tricosanoic acid (23:0me; Sigma, St. Louis, EUA) served as an internal standard. FA methyl esters (FAME) separation was performed by gas chromatography in a Thermo Scientific™ TRACE™ Ultra Gas Chromatographer (Thermo Scientific™, Waltham, MA, USA). The equipment had a flame ionization detector (FID), a split/split-less injector, and a fused silica capillary column CP-7420 (Select FAME, 100 m size, 0.25 mm of internal diameter, and 0.25 μm film thickness of the cyanopropyl stationary phase). The operational parameters were: the gas flow rates used were 1.2 mL min^−1^ for the carrier gas (H_2_), 30 mL min^−1^ for the make-up gas (N_2_), and 30 and 300 mL min^−1^ for the FID gas H_2_ and synthetic air, respectively. The injected sample volume was 1.0 µL, with a split injection ratio of 1:40. The column temperature was maintained at 165 °C for 18 min and then ramped to 235 °C (4 °C min^−1^) for 20 min. The injector and detector temperatures were kept at 230 °C and 250 °C, respectively.

We identified FAMEs by comparison of the retention times of the sample constituents with Sigma FAMEs. Retention times and peak areas were determined using the Chrom-Quest™ software (Thermo Fisher Scientific™, MA, USA). FA levels were calculated according to Visentainer (2012) [40]. FA contents in the diets and livers are expressed as mg/100 mg.

### 2.3. Gene Expression Measurement

We measured the mRNA expression of F4/80, interleukin 1β (IL-1β), interleukin 6 (IL-6), interleukin 10 (IL-10), and tumor necrosis factor-alpha (TNF-α) in the livers of mice from the C and S groups (day 0, 7, 14, 28, or 56). We also measured the liver expression of acetyl-CoA carboxylase 1 (ACC1) on day 56. Total RNA was extracted using Trizol reagent (Invitrogen Life Technologies, Waltham, MA, USA) and reverse-transcribed to cDNA (High-Capacity cDNA kit, Applied Biosystems, Foster USA, Waltham, MA, USA). Gene expression was evaluated by real-time PCR using SYBR Green as the fluorescent dye (Invitrogen Life Technologies, Waltham, MA, USA). We performed gene expression analysis using the ribosomal protein lateral stalk subunit P0 gene (Rplp0) as the internal control. The primer sequence was: F4/80 (NM_010130.4) sense CCTGAACATGCAACCTGCCAC, antisense GGGCATGAGCAGBCTGTAGGATC, IL-1β (NM_008361.4) sense GGCAGCTACCTGTGTCTTTCCC, antisense ATATGGGTCCGACAGCACGAG, IL-6 (NM_001314054.1) sense GGTAGCATCCATCATTTCTTTG, antisense, CGGAGAGGAGACTTCACAAGAG, TNF-α (NM_001278601.1) sense TCTTCTCATTCCTGCTTGTGGC, antisense CACTTGGTGGTTTGCTACGACG, IL-10 (NM_010548.2) sense TGCCAAGCCTTATCGGAAATG, antisense AAATCGATGACAGCGCCTCAG, *ACC1* (NM_133360.2) sense GAGAGGGGTCAAGTCCTTCC, antisense AAAACATCCACTTCCACACACGA, Rplp0 (NM_007475.5) sense CCACTTACTGAAAAGGTCAAGGC, antisense TGGTTGCTTTGGCGGGATTA.

### 2.4. Statistical Analysis

Results are presented as mean ± standard error, as analyzed by ANOVA (one-way) and Tukey’s post test. We compared each FA of the S group or C group using unpaired Student’s *t*-test. We performed Statistical analyses using the Graph-Pad Prism software. The 95% level of confidence (*p* < 0.05) was accepted for all comparisons.

## 3. Results

### 3.1. Food Intake, Body Weight, Liver Weight, Serum Glucose, Triacylglycerol, and Cholesterol

The changes from day 0 to day 56 for body weight (S Δ% = 37.1; C Δ% = 33.0), glucose (S Δ% = 6.8; C Δ% = 16.2), triacylglycerol (S Δ% = 78.5; C Δ% = 89.1), and cholesterol (S Δ% = 38.1; C Δ% = 44.3) did not differ between the S group and the C group. Food intake, liver weight, and relative liver weight were higher (*p* < 0.05) in the C group on day 56 (Table 2).

### 3.2. Saturated Fatty Acid (SFA) Composition

The S and C groups showed higher (*p* < 0.05) lauric acid (12:0), myristic acid (14:0), palmitic acid (16:0), stearic acid (18:0), and arachidic acid (20:0) on day 56 (day 56 vs. day 0). Heneicosanoic acid (21:0) increased (*p* < 0.05) and decreased (*p* < 0.05) between days 0 and 56 in the S and C groups, respectively (Table 3).

On day 56, the C group exhibited higher (*p* < 0.05) levels of arachidic acid (20:0) in comparison with the S group. On the other hand, on day 56, the C group showed lower (*p* < 0.05) levels of heneicosanoic acid (21:0), in comparison with the S group. The contents of lauric acid (12:0), myristic acid (14:0), palmitic acid (16:0), and stearic acid (18:0) did not differ in C vs. S group (Table 3).

### 3.3. Monounsaturated Fatty Acid (MUFA) Composition

The levels of palmitoleic acid (16:1n-7), 7-hexadenoic acid (16:1n-9), vaccenic acid (18:1n-7), oleic acid (18:1n-9), and gondoic acid (20:1n-9) increased (*p* < 0.05) for both groups (day 56 vs. day 0). Nervonic acid (24:1n-9) increased (*p* < 0.05) only for the C group but decreased (*p* < 0.05) in the S group (day 56 vs. day 0) (Table 4).

On day 56, the C group exhibited higher (*p* < 0.05) levels of palmitoleic acid (16:1n-7), 7-hexadenoic acid (16:1n-9), vaccenic acid (18:1n-7), oleic acid (18:1n-9), gondoic acid (20:1n-9), and nervonic acid (24:1n-9) in comparison with the S group (Table 4).

### 3.4. Polyunsaturated n-6 Fatty Acid (PUFA) Composition

Linoleic acid (18:2n-6), γ-linolenic acid (18:3n-6), 11,14-eicosadienoic acid (20:2n-6), and arachidonic acid (20:4n-6) increased *(p* < 0.05) in both groups (day 56 vs. day 0). On day 56, the C group exhibited lower (*p* < 0.05) levels of linoleic acid (18:2n-6), γ-linolenic acid (18:3n-6), and arachidonic acid (20:4n-6) in comparison with the S group. In contrast, the C group showed higher (*p* < 0.05) levels of 11,14-eicosadienoic acid (20:2n-6) on day 56 (Table 5).

### 3.5. Polyunsaturated n-3 Fatty Acid (PUFA) Composition

α-linolenic acid (18:3n-3), dihomo-α-linolenic acid (20:3n-3), eicosapentaenoic acid (EPA, 20:5n-3), and docosahexaenoic acid (DHA, 22:6n-3) increased (*p* < 0.05) in both groups (day 56 vs. day 0). On day 56, the C group exhibited higher (*p* < 0.05) levels of α-linolenic acid (18:3n-3), eicosapentaenoic acid (EPA, 20:5n-3), and docosahexaenoic acid (DHA, 22:6n-3) in comparison with the S group. However, dihomo-α-linolenic acid (20:3n-3) content was similar between the groups (Table 6).

### 3.6. Analysis of Fatty Acids (FAs) Family Composition and n-6:n-3ratio

The liver lipid deposition (calculated by the sum of all FAs) and liver deposition of SFA, MUFA, n-6 PUFA, and n-3 PUFA were intensified (*p* < 0.05) during the experimental period (day 0 vs. day 56) for both groups: SUM (S Δ% = 229.5; C Δ% = 272.3), SFA (S Δ% = 195.5; C Δ% = 204.6), MUFA (S Δ% = 689.5; C Δ% = 1072.4), n-6 PUFA (S Δ% = 131.3; C Δ% = 47.9), and n-3 PUFA (S Δ% = 59.4; C Δ% = 116.0) (Table 7).

On day 56, the C group showed a higher (*p* < 0.05) value for the sum of all fatty acids evaluated. The C group also exhibited higher (*p* < 0.05) levels of MUFA and n-3 PUFA. In contrast, the C group showed lower (*p* < 0.05) levels of n-6 PUFA, while the SFA levels were similar (C group vs. S group) (Table 7).

The n-6 PUFA:n-3 PUFA ratio increased (*p* < 0.05) in the S group (Δ% = 44.9) and decreased (*p* < 0.05) in the C group (Δ% = −31.7) from day 0. The C group showed a lower (*p* < 0.05) n-6 PUFA:n-3 PUFA ratio from day 7 until day 56, in comparison with the S group(Table 7).

### 3.7. Gene Expressions

**Figure 2:**
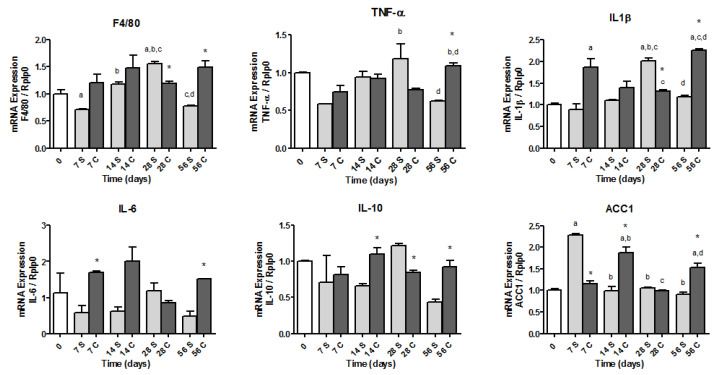
mRNA expression of inflammatory genes and acetyl-CoA carboxylase 1 (ACC1) in the livers of mice fed with a diet containing soybean oil (S group) or canola oil (C group) as a lipid source, at 0, 7, 14, 28, or 56 days after starting the diets. Abbreviations: TNF-α, tumor necrosis; IL, interleukin. Results are expressed as mean ± standard error. *p* < 0.05 as compared with day 0 ^a^, day 7 ^b^, day 14 ^c^, day 28 ^d^, and S group *.

The gene expression of IL-1β and ACC1 increased (*p* < 0.05) in the C group (day 56 vs. day 0). On day 56, the C group had higher (*p* < 0.05) gene expressions of F4/80, TNF-α, IL-1β, IL-6, IL-10, and ACC1, in comparison with the S group (Figure 2).

## 4. Discussion

The health benefits of canola oil, compared with soybean oil, are usually expected, as the former has a lower n-6:n-3 ratio [19]. However, canola oil’s potential beneficial effects still face many controversies [19,20,21,22,23,24,25,26,27,28,29,30,31,32,33,34,35,36], and little is known about its effects on liver FAs deposition and inflammation.

The levels of PUFAs and MUFAs, particularly the levels of α-linolenic acid and oleic acid in the livers of the C group reflected the lipid composition of canola oil in the diet. The C group showed lower liver concentrations of γ-linolenic acid and arachidonic acid (synthesized from linoleic acid), and higher liver levels of dihomo-α-linolenic acid, EPA, and DHA (synthesized from α-linolenic acid).

The total amount of FAs between days 0 and 56, in the livers of the S and C groups increased by 229.5% and 272.3%, respectively. The mechanisms by which a high-carbohydrate diet increases lipid deposition involve the intensification of the generation of acetyl-CoA from glucose. Acetyl-CoA activates the transcription factors sterol regulatory element-binding proteins (SREBP-1c) and carbohydrate response element binding protein (ChREBP), which regulate key genes involved in the lipid synthesis, such as ACC1 [41,42,43].

The higher concentrations of n-3 PUFAs in the livers of the C group could prevent liver DNL via downregulation of SREBP-1c and ChREBP gene expression and stimulation of FA oxidation [44,45]. However, in this study, the diet containing canola oil as a lipid source did not prevent liver lipid accumulation.

The higher liver lipid accumulation in livers from the C group could be explained by the greater amount of MUFAs in the C diet. In agreement with this affirmation, Duwaerts et al. [46] reported that an MUFA-enriched diet is more steatogenic than an SFA-enriched diet, particularly when combined with complex carbohydrates such as starch. In addition, mice fed with diets rich in oleic acid have shown high liver lipid deposition [47,48]. Oleic acid, the main MUFA in the C group’s diet and liver, promotes liver steatosis, oxidative stress, apoptosis, and the increased production of TNF-α and IL-6 [47,48,49].

Higher levels of n-3 PUFAs, such as EPA (20:5n-3) and DHA (22:6n-3), are expected to prevent liver inflammation, as they are precursors to anti-inflammatory mediators [50]. However, the livers of the C group exhibited higher inflammation, as suggested by the higher gene expressions of F4/80, TNF-α, IL1-β, and IL-6. The cytokines TNF-α, IL1-β, and IL-6 are involved in the inflammatory process by producing other cytokines that promote chronic inflammation [51]. Moreover, F4/80 is a marker of the liver recruitment of macrophages from resident Kupffer cells and circulating monocytes, which play a central role in the progression of NAFLD [52]. Furthermore, the simultaneous increase of gene expression of IL-10 (an anti-inflammatory cytokine) represents a negative feedback mechanism, in an attempt to protect the liver against an exacerbated inflammatory response [53].

In agreement with our results, other reports have also demonstrated that canola oil promotes higher oxidative stress and inflammation than soybean oil, safflower oil, or flax oil [30,54]. It is likely that other components of canola oil, which were not investigated in this study, could influence oxidative stress and inflammation; for example, the production of cyclic FAs monomers and/or the loss of phenolic compounds during the industrial refining of canola oil [55,56,57].

The main limitation of this investigation was the restricted time period of evaluation (56 days). Additional limitations of the study included a reduced number of biomarkers of lipogenesis and inflammation. Despite these limitations, we can conclude that the replacement of soybean oil by canola oil as a lipid source did not prevent the liver FA accumulation and inflammation induced by a high-carbohydrate diet in mice.

## Figures and Tables

**Table 1:** Fatty acid composition (mg/g) of the American Institute of Nutrition (AIN-93-M) diet with soybean oil (S group) or canola oil (C group) as a lipid source.

Fatty Acids	S Group	C Group
Palmitic acid (16:0)	70.01 ± 1.12	40.24 ± 0.37 *
Stearic acid (18:0)	26.25 ± 0.40	20.19 ± 0.13 *
Oleic acid (18:1n-9)	150.88 ± 2.24	420.41 ± 3.85 *
Vaccenic acid (18:1n-7)	8.34 ± 0.16	21.52 ± 0.23 *
Linoleic acid (18:2n-6)	303.97 ± 4.46	136.98 ± 1.19 *
α-Linolenic acid (18:3n-3)	31.27 ± 0.46	61.43 ± 0.72 *
SFA	96.27 ± 1.53	60.43 ± 0.51 *
MUFA	159.23 ± 2.40	441.93 ± 4.09 *
PUFA	336.79 ± 4.94	198.41 ± 1.92 *
n-6	305.52 ± 4.47	136.98 ± 1.19 *
n-3	31.27 ± 0.46	61.43 ± 0.72 *
n-6/n-3	9.76 ± 0.00	2.58 ± 0.00 *

Results are expressed as mean ± standard error. Abbreviations: SFA, total saturated fatty acids; MUFA, total monounsaturated fatty acids; PUFA, total polyunsaturated fatty acids; n-6, omega-6 PUFAs; n-3, omega-3 PUFAs. *p* < 0.05 compared with S group *.

**Table 2:** Food intake, body weight, liver weight, relative liver weight, serum glucose, triacylglycerol, and cholesterol, of mice fed with a diet containing soybean oil (S group) or canola oil (C group) as a lipid source, at 0, 7, 14, 28, or 56 days after starting the diets.

		Day 0	Day 7	Day 14	Day 28	Day 56	∆%
Food intake (g/day)	S group	-	9.54 ± 0.97	10.10 ± 0.57	9.10 ± 0.40	7.71 ± 0.13	-
	C group	-	10.46 ± 0.74	8.83 ± 0.62	9.04 ± 0.60	8.35 ± 0.14 *	-
Body weight (g)	S group	34.78 ± 0.42	36.38 ± 1.16	40.55 ± 1.05	51.58 ± 2.41 ^a,b,c^	47.70 ± 2.03 ^a,b,c^	37.1
	C group		37.28 ± 0.93	44.46 ± 1.23 *	52.66 ± 3.92 ^a,b^	46.27 ± 3.42 ^a^	33.0
Liver weight (g)	S group	1.42 ± 0.02	1.33 ± 0.16	1.45 ± 0.03	1.74 ± 0.07 ^a,b^	1.51 ± 0.04	6.3
	C group		1.36 ± 0.10	1.68 ± 0.09	1.97 ± 0.22 ^a,b^	1.73 ± 0.03 *	21.8
Relative liver weight (g/100g)	S group	4.10 ± 0.06	3.67 ± 0.51	3.58 ± 0.03	3.40 ± 0.11	3.24 ± 0.05 ^a^	−20.9
	C group		3.64 ± 0.22	3.63 ± 0.06	3.72 ± 0.21	3.49 ± 0.09 *	−14.8
Glucose (mg/dL)	S group	108.70 ± 6.43	104.60 ± 11.80	92.26 ± 4.71	91.96 ± 8.15	116.16 ± 7.09	6.8
	C group		96.26 ± 4.13	100.23 ± 7.48	101.20 ± 13.41	126.30 ± 9.06	16.2
Triacylglycerol (mg/dL)	S group	90.18 ± 11.24	173.16 ± 14.22 ^a^	212.08 ± 39.13 ^a^	167.33 ± 13.47	160.99 ± 19.02 ^a^	78.5
	C group		248.22 ± 21.37 ^a,^*	315.15 ± 57.84 ^a^	237.90 ± 18.22 ^a,^*	170.55 ± 11.04 ^c^	89.1
Cholesterol (mg/dL)	S group	132.38 ± 5.87	157.00 ± 8.34	124.60 ± 11.40	163.20 ± 11.32	182.86 ± 9.22 ^a,c^	38.1
	C group		151.60 ± 13.81	131.83 ± 20.55	199.20 ± 15.87 ^a,c^	191.13 ± 9.69 ^a,c^	44.3

Results are expressed as mean ± standard error. *p* < 0.05 as compared with day 0 ^a^, day 7 ^b^, day 14 ^c^, and day 28, and the S group *. ∆%: percentage change (day 0 vs. day 56).

**Table 3:** Saturated fatty acid (SFA) composition (mg/100 g of sample) in the livers of mice fed with a diet containing soybean oil (S group) or canola oil (C group) as a lipid source, at 0, 7, 14, 28, or 56 days after starting the diets.

SFA		Day 0	Day 7	Day 14	Day 28	Day 56	∆%
Lauric acid (12:0)	S group	1.37 ± 0.07	4.23 ± 0.81 ^a^	4.27 ± 0.24 ^a^	2.21 ± 0.08 ^b,c^	2.88 ± 0.43 ^a^	110.2
	C group		5.53 ± 0.37 ^a^	2.33 ± 0.12 ^a,b,^*	2.06 ± 0.06 ^b^	2.40 ± 0.30 ^a,b^	75.1
Myristic acid (14:0)	S group	6.36 ± 0.39	52.58 ± 12.98 ^a^	26.65 ± 1.48 ^a,b^	22.49 ± 0.57 ^a,b^	33.53 ± 0.61 ^a,b^	427.2
	C group		36.02 ± 2.38 ^a^	26.58 ± 2.53 ^a^	34.58 ± 1.13 ^a,^*	37.85 ± 3.08 ^a,c^	495.1
Palmitic acid (16:0)	S group	451.55 ± 31.74	1176.55 ± 68.73 ^a^	1267.90 ± 48.82 ^a^	1245.10 ± 13.58 ^a^	1567.95 ± 44.03 ^a,b,c,d^	247.2
	C group		1453.63 ± 4.10 ^a,^*	1312.49 ± 62.84 ^a^	1735.27 ± 12.6 ^a,b,c,^*	1644.88 ± 37.52 ^a,b,c^	264.2
Stearic acid (18:0)	S group	219.57 ± 16.4	447.56 ± 25.02 ^a^	453.21 ± 11.55 ^a^	404.55 ± 9.46 ^a^	411.70 ± 12.85 ^a^	87.5
	C group		469.20 ± 0.59 ^a^	464.50 ± 16.42 ^a^	469.79 ± 2.88 ^a,^*	391.29 ± 8.58 ^a,b,c,d^	78.2
Arachidic acid (20:0)	S group	3.19 ± 0.19	10.30 ± 0.98 ^a^	6.70 ± 0.72 ^a,b^	7.64 ± 0.21 ^a^	12.15 ± 0.28 ^a,c,d^	280.8
	C group		17.37 ± 0.60 ^a,^*	10.37 ± 0.48 ^a,b,^*	9.88 ± 0.39 ^a,b,^*	16.84 ± 1.31 ^a,c,d,^*	427.8
Heneicosanoic acid (21:0)	S group	6.35 ± 0.41	9.28 ± 0.17 ^a^	3.77 ± 0.59 ^a,b^	5.95 ± 0.09 ^b,c^	8.09 ± 0.45 ^a,c,d^	27.4
	C group		10.79 ± 0.52 ^a^	4.64 ± 0.14 ^a,b^	3.56 ± 0.15 ^a,b,^*	4.17 ± 0.17 ^a,b,^*	−34.3

Results are expressed as mean ± standard error of three replicates for each group. *p* < 0.05 as compared with day 0 ^a^, day 7 ^b^, day 14 ^c^, and day 28 ^d^, and S group *. ∆%: percentage change (day 0 vs. day 56).

**Table 4:** Monounsaturated fatty acid (MUFA) composition (mg/100 g of sample) in the livers of mice fed with a diet containing soybean oil (S group) or canola oil (C group) as a lipid source, at 0, 7, 14, 28, or 56 days after starting the diets.

MUFA		Day 0	Day 7	Day 14	Day 28	Day 56	∆%
Palmitoleic acid (16:1n-7)	S group	20.82 ± 1.45	121.40 ± 6.32 ^a^	186.67 ± 10.21 ^a^	205.95 ± 4.01 ^a,b,c^	308.54 ± 6.63 ^a,b,c,d^	1381.9
	C group		168.55 ± 1.01 ^a,^*	183.85 ± 12.39 ^a^	265.35 ± 9.80 ^a,b,c,^*	355.62 ± 10.61 ^a,b,c,d,^*	1608.0
7-hexadecanoic acid (16:1n-9)	S group	7.84 ± 0.42	21.42 ± 1.00 ^a^	21.63 ± 1.50 ^a^	26.53 ± 0.72 ^a,b,c^	42.50 ± 1.18 ^a,b,c,d^	442.0
	C group		40.48 ± 0.62 ^a,^*	48.13 ± 1.35 ^a,^*	157.33 ± 8.77 ^a,b,c,^*	69.90 ± 2.18 ^a,b,c,d,^*	791.5
Vaccenic acid (18:1n-7)	S group	31.72 ± 2.24	92.76 ± 3.17 ^a^	116.16 ± 4.76 ^a^	140.88 ± 1.90 ^a,b,c^	225.60 ± 9.28 ^a,b,c,d^	611.2
	C group		133.74 ± 0.59 ^a,^*	144.52 ± 6.14 ^a,^*	250.94 ± 1.55 ^a,b,c,^*	271.54 ± 6.02 ^a,b,c,d,^*	756.0
Oleic acid (18:1n-9)	S group	225.55 ± 13.84	1283.73 ± 157.30 ^a^	1197.56 ± 71.24 ^a^	1222.64 ± 18.93 ^a^	1789.55 ± 49.59 ^a,b,c,d^	693.4
	C group		1633.75 ± 12.65 ^a^	1766.14 ± 77. 38 ^a,^*	3402.46 ± 5.38 ^a,b,c,^*	2814.65 ± 80.90 ^a,b,c,d,^*	1147.9
Gondoic acid (20:1n-9)	S group	3.31 ± 0.22	9.00 ± 0.79 ^a^	12.70 ± 0.78 ^a,b^	12.98 ± 0.21 ^a,b^	23.78 ± 1.01 ^a,b,c,d^	618.4
	C group		16.37 ± 0.13 ^a,^*	16.30 ± 0.92 ^a,^*	27.06 ± 0.19 ^a,b,c,^*	35.66 ± 2.48 ^a,b,c,d,^*	977.3
Nervonic acid (24:1n-9)	S group	14.23 ± 0.72	12.11 ± 0.08	12.70 ± 2.16	12.91 ± 0.05	12.25 ± 0.60	−13.9
	C group		20.01 ± 1.18 ^a,^*	17.01 ± 0.45 ^b^	16.50 ± 0.27 *	21.67 ± 0.53 ^a,c,d,^*	52.2

Results are expressed as mean ± standard error of three replicates for each group. *p* < 0.05 as compared with day 0 ^a^, day 7 ^b^, day 14 ^c^, and day 28 ^d^, and S group *. ∆%: percentage change (day 0 vs. day 56).

**Table 5:** Polyunsaturated n-6 fatty acid (n-6 PUFA) composition (mg/100g of sample) in the livers of mice fed with a diet containing soybean oil (S group) or canola oil (C group) as a lipid source, at 0, 7, 14, 28, or 56 days after starting the diets.

n-6 PUFA		Day 0	Day 7	Day 14	Day 28	Day 56	∆%
Linoleic acid (18:2n-6)	S group	525.33 ± 32.43	1534.81 ± 153.60 ^a^	1411.13 ± 67.48 ^a^	964.554 ± 11.09 ^a,b,c^	1330.23 ± 43.46 ^a^	153.2
	C group		1731.06 ± 25.28 ^a^	827.076 ± 33.06 ^a,b,^*	677.56 ± 9.56 ^a,b,c,^*	830.28 ± 30.48 ^a.b,d,^*	58.0
γ-linolenic acid (18:3n-6)	S group	14.26 ± 0.82	30.98 ± 1.64 ^a^	32.65 ± 1.95 ^a^	24.18 ± 0.58 ^a,c^	33.77 ± 1.93 ^a,d^	136.8
	C group		50.34 ± 2.50 ^a,b,^*	21.85 ± 0.85 ^a,b,^*	17.18 ± 0.44 ^b,^*	21.93 ± 1.89 ^a,b,^*	53.7
11,14-eicosadienoic acid (20:2n-6)	S group	1.26 ± 0.10	3.81 ± 0.07 ^a^	1.68 ± 0.23 ^b^	6.62 ± 0.26 ^a,b,c^	5.36 ± 0.20 ^a,b,c,d^	325.3
	C group		8.37 ± 0.22 ^a,^*	10.50 ± 0.19 ^a,b,^*	16.37 ± 0.33 ^a,b,c,^*	13.69 ± 0.42 ^a,b,c,d,^*	986.5
Arachidonic acid (20:4n-6)	S group	259.06 ± 19.06	435.25 ± 4.67 ^a^	454.75 ± 4.23 ^a^	460.06 ± 10.96 ^a^	479.56 ± 18.85 ^a^	85.1
	C group		416.30 ± 0.47 ^a,^*	395.02 ± 12.34 ^a,^*	349.73 ± 5.49 ^a,b,^*	316.38 ± 3.57 ^a,b,c,^*	22.1

Results are expressed as mean ± standard error of three replicates for each group. *p* < 0.05 as compared with day 0 ^a^, day 7 ^b^, day 14 ^c^, and day 28 ^d^, and S group *. ∆%: percentage change from day 0.

**Table 6:** Polyunsaturated n-3 fatty acid (n-3 PUFA) composition (mg/100g of sample) in the livers of mice fed with a diet containing soybean oil (S group) or canola oil (C group) as a lipid source, at 0, 7, 14, 28, or 56 days after starting the diets.

n-3 PUFA		Day 0	Day 7	Day 14	Day 28	Day 56	∆%
α-linolenic acid (18:3n-3)	S group	16.37 ± 0.96	57.46 ± 4.27 ^a^	55.85 ± 3.97 ^a^	34.53 ± 0.66 ^a,b,c^	42.33 ± 1.83 ^a,b,c^	158.5
	C group		82.63 ± 1.21 ^a,^*	47.12 ± 2.13 ^a,b^	42.73 ± 0.99 ^a,b,^*	74.23 ± 6.04 ^a,c,d,^*	353.4
Dihomo-α-linolenic acid (20:3n-3)	S group	15.23 ± 1.07	26.60 ± 0.34 ^a^	30.45 ± 0.33 ^a^	36.16 ± 0.75 ^a,b^	44.19 ± 3.48 ^a,b,c,d^	190.1
	C group		28.09 ± 1.10 ^a^	36.35 ± 1.43 ^a,b,^*	47.69 ± 0.67 ^a,b,c,^*	49.09 ± 1.44 ^a,b,c^	222.3
Eicosapentaenoic acid (20:5n-3)	S group	3.97 ± 0.34	3.87 ± 0.61 ^a^	10.39 ± 1.15 ^a^	8.97 ± 0.21 ^a^	11.71 ± 0.56 ^a,b^	194.9
	C group		17.00 ± 0.49 ^a,^*	27.35 ± 0.97 ^a,b,^*	36.70 ± 0.99 ^a,b,c,^*	48.11 ± 1.01 ^a,b,c,d,^*	1111.8
Docosahexaenoic acid (22:6n-3)	S group	188.79 ± 12.06	228.63 ± 3.43 ^a^	283.76 ± 7.76 ^a,b^	273.89 ± 6.12 ^a,b^	259.47 ± 5.15 ^a^	37.4
	C group		297.89 ± 5.55 ^a,^*	314.45 ± 12.18 ^a^	295.23 ± 2.63 ^a,^*	313.31 ± 3.03 ^a,^*	65.9

Results are expressed as mean ± standard error of three replicates for each group. *p* < 0.05 as compared with day 0 ^a^, day 7 ^b^, day 14 ^c^, and day 28 ^d^, and S group *. ∆%: percentage change from day 0.

**Table 7:** Fatty acids family composition (mg/100 g of sample) and n-6:n-3 PUFA ratio in the livers of mice fed with a diet containing soybean oil (S group) or canola oil (C group) as a lipid source, at 0, 7, 14, 28, or 56 days after starting the diets.

Fatty Acids		Day 0	Day 7	Day 14	Day 28	Day 56	∆%
SFA	S group	690.66 ± 49.46	1694.58 ± 110.10 ^a^	1767.14 ± 61.13 ^a^	1691.58 ± 22.29 ^a^	2041.59 ± 56.74 ^a,b,d^	195.5
	C group		1998.79 ± 8.37 ^a^	1824.68 ± 82.56 ^a^	2259.49 ± 13.78 ^a,b,c,^*	2104.20 ± 51 ^a,c^	204.6
MUFA	S group	305.02 ± 19.07	1555.54 ± 167.30 ^a^	1551.91 ± 87.71 ^a^	1625.98 ± 25.26 ^a^	2408.39 ± 64.61 ^a,b,c,d^	689.5
	C group		2017.12 ± 12.99 ^a,^*	2179.19 ± 99.01 ^a,^*	4124.39 ± 5.66 ^a^^,b,c,^*	3576.26 ± 103.00 ^a,b,c,d,^*	1072.4
PUFA	S group	1024.32 ± 66.86	2325.86 ± 168.70 ^a^	2280.69 ± 75.50 ^a^	1809.00 ± 18.63 ^a,b,c^	2206.65 ± 62.31 ^a^	115.4
	C group		2631.73 ± 27.35 ^a^	1679.74 ± 63.16 ^a,b,^*	1483.22 ± 17.60 ^a,b,^*	1667.06 ± 36.96 ^a,b,^*	62.7
n-6	S group	799.33 ± 52.42	2004.87 ± 160.00 ^a^	1900.23 ± 71.01 ^a^	1455.42 ± 13.77 ^a,b,c^	1848.93 ± 54.55 ^a^	131.3
	C group		2206.09 ± 26.50 ^a^	1254.44 ± 46.44 ^a,b,^*	1060.85 ± 13.96 ^a,b,c,^*	1182.30 ± 31.24 ^a,b,^*	47.9
n-3	S group	224.38 ± 14.44	320.99 ± 8.66 ^a^	380.46 ± 8.84 ^a^	353.57 ± 6.57 ^a^	357.72 ± 9.11 ^a^	59.4
	C group		426.23 ± 3.31 ^a,^*	425.30 ± 16.72 ^a^	422.37 ± 4.15 ^a,^*	484.75 ± 6.79 ^a,b,c,d,^*	116.0
SUM	S group	2020.12 ± 135.40	5408.39 ± 272.70 ^a^	5599.73 ± 38.21 ^a^	5126.56 ± 51.41 ^a^	6656.63 ± 180.60 ^a,b,d^	229.5
	C group		6647.63 ± 38.21 ^a,^*	5683.60 ± 244.70 ^a,b^	7867.11 ± 28.08 ^a,b^^,c,^*	7522.60 ± 132.00 ^a,b,c,^*	272.3
n-6/n-3	S group	3.56 ± 0.00	6.22 ± 0.33 ^a^	4.99 ± 0.16 ^a,b^	4.11 ± 0.06 ^b,c^	5.16 ± 0.08 ^a,b,d^	44.9
	C group		5.18 ± 0.06 ^a,^*	2.95 ± 0.00 ^a,b,^*	2.51 ± 0.01 ^a,b,c,^*	2.43 ± 0.04 ^a,b,c,^*	−31.7

Results are expressed as mean ± standard error of three replicates for each group. Abbreviations: SFA, total saturated fatty acids; MUFA, total monounsaturated fatty acids; PUFA, total polyunsaturated fatty acids; SUM, the sum of all fatty acids evaluated. *p* < 0.05 as compared with 0 ^a^, day 7 ^b^, day 14 ^c^, and day 28 ^d^; and S group *. ∆%: percentage change from day 0.
